# Evaluation and significance of a digital assistant for patient history-taking and physical examination in telemedicine

**DOI:** 10.1093/oodh/oqae008

**Published:** 2024-02-02

**Authors:** Neha Verma, Bimal Buch, R S Pandya, Radha Taralekar, Ishita Masand, Hardik Rangparia, J M Katira, Soumyadipta Acharya

**Affiliations:** Division of Biomedical Informatics & Data Science, Johns Hopkins University School of Medicine, 2024 East Monument St. S 1-200, Baltimore, MD 21205, USA; Intelehealth, 14A Shreeji Arcade, Panchpakhadi, Thane 400602, Maharashtra, India; Intelehealth, 14A Shreeji Arcade, Panchpakhadi, Thane 400602, Maharashtra, India; Intelehealth, 14A Shreeji Arcade, Panchpakhadi, Thane 400602, Maharashtra, India; Division of Biomedical Informatics & Data Science, Johns Hopkins University School of Medicine, 2024 East Monument St. S 1-200, Baltimore, MD 21205, USA; District Health Department, Gibbson Middle School, Opposite Railway Station, Health Branch, Morbi 363641, Gujarat, India; District Health Department, Gibbson Middle School, Opposite Railway Station, Health Branch, Morbi 363641, Gujarat, India; Center for Bioengineering Innovation & Design, Johns Hopkins University, Clark Hall, Suite 208, 3400 Charles St, Baltimore, MD, USA

**Keywords:** telemedicine, task-shifting, digital assistant, mhealth, ehealth, frontline health worker

## Abstract

**Introduction:**

Assisted history-taking systems can be used in provider-to-provider teleconsultations to task-shift the collection of evidence-based medical history and physical exam information to a frontline health worker. We developed such a task-shifting digital assistant, called ‘Ayu’, for nurses in rural India to collect clinical information from a patient and share it with a remote doctor to arrive at an accurate diagnosis and triage decision.

**Materials & Methods:**

We evaluated the ability of the task-shifting digital assistant to collect a comprehensive patient history by using 190 standardized patient case studies and evaluating the information recall of the assistant by a skilled clinician. Following this, we tested the ability of nurses to use the system by training and evaluating the system’s accuracy when used by 19 nurses in rural Gujarat, India. We also measured the diagnostic and triage accuracy based on the generated history note. Finally, we evaluated the system’s acceptability by using the Technology Acceptance Model framework.

**Results:**

Ayu could capture 65% of patient history information and 42% of physical exam information from patient case studies. When used by nurses, the mean accuracy of the generated clinical note was 7.71 ± 2.42. Using the information collected by a nurse using Ayu, a primary care physician could arrive at the correct diagnosis in 74% of cases, and correct triage decision in 88% of cases. Overall, we saw a high acceptability from nurses to use the system.

**Conclusions:**

Ayu can capture an acceptable proportion of clinical information and can aid in collecting an evidence-based medical history by task-shifting some of the early investigational steps. Further development of Ayu to increase its information retrieval ability and ease of use by health workers is needed.

## INTRODUCTION

In a telemedicine encounter, the patient-reported history is often the only clinical information available to the physician to make a diagnosis. Never has the age-old saying by William Osler been more accurate than in telemedicine, ‘Listen to the patient. He is telling you the diagnosis’. Several studies have shown that incomplete history taking is a leading factor contributing to diagnostic errors in telemedicine [1–3]. Despite the advances made in diagnostic testing, a well-taken patient history and physical examination are still the primary sources of reliable clinical information for making a diagnosis and management plan [4]. Various studies have shown that the medical history alone can lead to a diagnosis in 59–80% of cases, the physical exam can lead to a diagnosis in 8–20% of cases and investigations in 8–20% of cases [5–7].

Over the past decade, several computer-assisted history-taking systems have been developed that systematically elicit patient history, emulating the rules-based algorithms of how clinicians are taught to gather information in a patient interview [8,9]. Typical advantages of the use of computer-assisted history taking reported in the literature are: (1) improvement in clinical documentation while reducing the time spent by a provider in documentation, (2) improved face time with the patient, (3) improved ability to collect more comprehensive and relevant information, (4) overall improvement in the quality of information gathered and (5) reduction in variability in the history-taking process by making sure critical questions are never omitted [8–11]. Several studies have reported high levels of patient satisfaction with using electronic tools for assisted history-taking [12–14].

A study by Kantarcigil *et al*. [12] demonstrated the effectiveness of the use of an assisted history-taking system for the assessment of dysphagia in a telemedicine setting. The low use of assisted history-taking systems in telemedicine combined with their known effectiveness [8], potential benefits in a telemedicine setting in a developing country and our experiences developing and implementing telemedicine technology in India [15] led us to develop ‘Ayu’, a tool for assisted history-taking prior to a teleconsultation with a doctor.

Assisted history-taking systems have been developed for several clinical use cases and often for specific domains such as emergency medicine [13], prenatal care [16] or specific illnesses such as cardiovascular disease [17], type 2 diabetes [18], gastrointestinal disease [19], obesity [20] and Chronic Obstructive Pulmonary Disease (COPD) [21]. Very few are developed for general medicine [11]. The systems can vary from simple (32 questions) to complex (6000+ questions). Ayu was developed to collect information from patients reporting to outpatient primary care clinics in India but can be generalized to other developing countries with similar etiology and disease epidemiology.

We developed Ayu for frontline health workers (FHWs) such as community health workers (CHWs), nurses or midwives to elicit a patient’s history and collect data from basic physical exams prior to a teleconsultation with a remote doctor. We hypothesized that this task-shifting of history-taking to a health professional at the patient’s side allows the remote doctor to provide better quality care, saves the total time taken by the doctor and improves the overall adherence to evidence-based medicine. It is a rules-based algorithm that takes the user through a sequence of steps for (1) entering basic vital signs information, (2) selecting from a list of 52 presenting complaints, (3) answering a questionnaire for each presenting complaint, (4) answering questions related to any associated symptoms, (5) entering the patient’s medical history & social history, (6) entering the patient’s family history, (7) prompting the user to conduct relevant physical exams triggered by the responses in the history-taking sections, (8) allowing the user to upload patient images as well as (9) any reports of investigations or imaging to finally (10) generate the output clinical note.

Not all aspects of clinical data collection are task-shifted using Ayu, bearing in mind the ability of the frontline health worker to collect this information reliably. For example, exams such as collecting heart sounds or lung sounds using a stethoscope, conducting a vaginal or scrotal examination and history-taking for sensitive topics such as psychiatric conditions are not task-shifted. The focus is on task-shifting processes that require minimal training for the FHW. The questions do not intend to replace a doctor–patient conversation but to task-shift some of the early investigational steps. We have previously published a detailed paper on the process of designing and developing Ayu and its applicability in the context of provider-to-provider telemedicine programs [22].

The objectives of this study were to evaluate the following aspects of Ayu:

1. Information retrieval ability: To evaluate the extent to which the protocol can gather essential clinical information, i.e. the comprehensiveness and completeness of history-taking achieved by the digital tool (Ayu) and its impact on the remote physicians’ ability to diagnose and triage the patient correctly.
2. Fidelity of use by nurses: To evaluate whether nurses can use a digital tool (Ayu) with protocols for history-taking and physical examination correctly.
3. Acceptability: To evaluate the acceptability of nurses to use a digital tool (Ayu) in their daily workflows.

## MATERIALS & METHODS

### Intelehealth, an open source telemedicine software, and Ayu, a history-taking digital assistant

**Figure 1:**
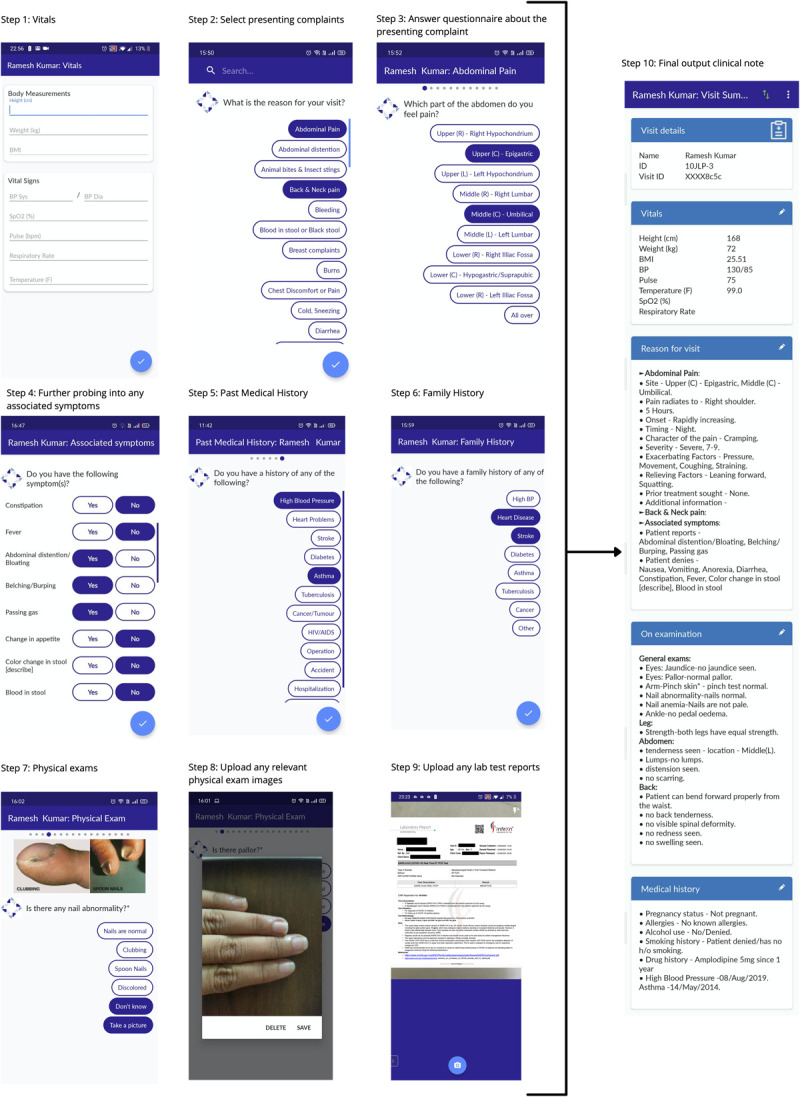
Flow of Ayu along with an example output.

Our study team has developed an open source digital public good telemedicine software called Intelehealth [15,22]. This telemedicine software has embedded within it a digital assistant called Ayu, which was also developed by our study team [22]. The software supports provider-to-provider teleconsults. The Intelehealth software consists of a mobile app and web portal via which frontline health providers can schedule video and audio consultations with remote specialized health providers to facilitate care for patients that they would otherwise have referred. The software platform is built for use in resource-limited settings—it is cloud-based, easy-to-use, works in low-bandwidth settings, is available in multiple languages, works on simple Android mobile devices and is distributed free and open source as a digital public good. The patient data captured during visits is securely stored in an electronic medical records database and can be visualized via data dashboards. The software platform also integrates with third party point-of-care diagnostic devices to measure parameters like blood pressure, blood sugar, electrocardiogram (ECG) readings, malaria diagnostics tests, blood tests and urine analysis. It has a FHIR API (Fast Healthcare Interoperability Resources Application Programming Interface) and an HL7 API (Health Level 7 Application Programming Interface) to enable health data exchange and is interoperable with other digital public goods such as OpenMRS and DHIS2 (District Health Information Software 2). It can be used to manage a wide spectrum of health issues related to reproductive, maternal and child health, infectious diseases, non-communicable diseases and primary and specialist care, with a particular focus on women’s health issues. The source code is available at https://github.com/intelehealth and a demo version of the application is available on the Google Play Store at https://play.google.com/store/apps/details?id=org.intelehealth.app,

Ayu, an integral component of the Intelehealth software, helps to shift the crucial task of clinical history-taking from a qualified doctor to that of a semi-skilled health worker, thereby supporting local health workers such as nurses/midwives and community health workers to provide evidence-based health services. Ayu covers 500+ history questions and 100+ physical exams. It is delivered through the Intelehealth mobile app interface for the end user. Figure 1 shows the system flow along with an example output. The version of Ayu used in this study had 52 structured clinical data collection checklists based on a patient’s presenting complaints. For example, there are essential checklists for history taking of a patient presenting with Fever, Cough, Abdominal Pain, Headache and many more. Based on the presenting complaint, Ayu prompts additional questions such as the site of the symptom, onset, duration and aggravating and relieving factors. It also prompts relevant questions related to the family history, social and occupational history, allergies, current medications, etc.

### Information retrieval ability

We selected 190 standardized patient case studies representing patients reporting to a primary care clinic in India. These included commonly seen as well as uncommonly seen cases. We also included some cases with atypical presentations of a common illness. We also included patient cases with tertiary care issues. While these patients would not likely be treated at a primary care facility (instead, be referred to a higher level facility), it is typical to see patients with advanced health issues first presenting at a primary care clinic in rural areas. We included cases from a range of medical specialties. A study team member (I.M.) selected these cases from various medical textbooks and teaching cases for medical students. The study team member (I.M.) was blinded to the content of the Ayu protocols so as not to bias the case selection process.

Another study team member (R.P.) used Ayu and followed the protocols to generate a history note and physical exam note from the standard patient case study. The study team member (R.P.) answered all the questions prompted by Ayu using the information provided in the case. If a question prompted by the protocol had no available answer in the case study, the user skipped the question. If a question prompted by Ayu was deemed ‘irrelevant’ to the case, the study team member (R.P.) skipped the question. Ayu contains a free-text section to enter information shared by the patient that does not fit into the questioning. However, the study team member (R.P.) did not use this section and entered no free-text information. The study team member (R.P.) only entered information in response to a direct question into Ayu. This clinical note generated by Ayu from the case study was uploaded to a web server, an Amazon Web Services (AWS) EC2 instance where the Intelehealth application was hosted. This process simulated the environment of a community health worker collecting information from a patient. This study team member (R.P.) was not involved in developing Ayu or selecting case studies to eliminate bias.

A third study team member (R.T.) analyzed the uploaded clinical notes and compared them to the original case using qualitative data analysis software (MaxQDA) through a deductive coding approach. Data presented in the case and captured in the generated clinical note was coded as ‘Captured information’ (true positives). Data present in the case but not captured in the general clinical note was coded as ‘Missed information’ (false negatives). The protocol aims to collect as much information as possible and at least all necessary information. However, some may be missed. These false negatives should be minimized but are unavoidable since many physical examinations cannot be task-shifted to the FHW.

The study team member (R.T.) noted the likely diagnosis and triage decision based on the generated clinical note. This diagnosis and triage decision was compared to the correct diagnosis and triage decision (treat remotely, urgent referral or non-urgent referral) by the same team member (R.T.). The study team member (R.T.) was blinded to the case study’s correct diagnosis and triage decision. While conducting the comparison, the study team member (R.T.) also recorded descriptive notes and observations about the protocols. These notes were later analyzed and discussed by the entire study team to generate critical insights on the improvement of the digital assistant.

Each of the study team members was assigned a specific role and had no contact or discussion with the other team members to avoid any bias. The first study team member (I.M.) selected medical cases and the second study team member (R.P.) used these selected medical cases to run them through Ayu; the third study team member (R.T.) compared the Ayu-captured medical cases with the original selected medical cases. Blinding of data collectors and outcome analyzer is crucial to ensure unbiased ascertainment of outcomes.

We analyzed the following data points for the history and physical examination sections of the generated note:

1. **Recall (Sensitivity):** Proportion of information from the case study captured in the generated clinical note. The ‘Recall score’ measures the overall sensitivity of the data collection protocol. The higher the recall, the better the information retrieval ability of Ayu. At the very least, the recall score should be such that all necessary and sufficient information is captured for the remote doctor to arrive at a differential diagnosis.
2. **Correct diagnosis rate:** Percentage of cases that could be correctly diagnosed based on the general clinical note alone
3. **Correct triage rate:** Percentage of cases that could be correctly triaged based on the general clinical note alone

Thus, we compared the system’s performance (generated clinical note) against a reference standard (case study). Recall and precision are commonly used to evaluate information retrieval in knowledge-based systems. Notably absent in our analysis was the calculation of precision or the positive predictive value of the protocol. The precision score was difficult to calculate since we used standardized patient case studies. Any additional questions prompted by Ayu, for which there was no information in the patient case, could be deemed ‘irrelevant’ or ‘relevant’, but this would largely depend on the reviewer’s clinical judgment. In the future, we plan to identify the precision using more rigorous methods such as retrospective chart review of a history note generated by Ayu and its comparison with a history note generated by an expert. However, since this was a preliminary feasibility study, we excluded the precision score from the analysis due to the inherent subjectivity in its measurement. Recall is the most critical measure of information retrieval for Ayu. The disadvantage of irrelevant questions is that the output clinical note would be filled with information not relevant to the diagnosis and would add to the doctor’s cognitive load and create some user irritation. However, in comparing whether we should place a greater value on precision or recall, missed information (a false negative) was more concerning than additional irrelevant information (a false positive) while making a diagnosis. Since Ayu intends to be a preliminary data collection step, it must capture all relevant information, i.e. sensitivity is more important than positive predictive value.

In the first phase of the study, a single team member, a trained physician, used Ayu to generate patient records. To understand the ability of our target user group (frontline health workers) to follow the same protocols in a high-fidelity manner, we conducted the second phase of the study with nurses.

### Fidelity of use by nurses

These nurses were selected from a site where the Intelehealth solution was going to be deployed in two blocks of Morbi, Gujarat, India. The District Health administration of Morbi was implementing a telemedicine program that would use a white-labeled version of the default Intelehealth software used in the first phase of the study, re-branded by the district administration as ‘MyTeleDoc’. The MyTeleDoc version included the same protocols from Ayu that were used in the first phase of the study.

#### Study site

Morbi is a relatively new district in central Gujarat, formed in the year 2013, and was earlier a part of Rajkot district. It has five blocks—Morbi, Maliya, Tankara, Vankaner and Halvad. As per the data shared by the district health administration, the district has 1 District Hospital, 5 Community Health Centers, 30 Primary Health Centers and 198 Sub-centers (which were undergoing an upgradation process to Health and Wellness Centers) catering to a total population of 10,13 ,952. Based on the primary data collected from the district health administration, we found that there is a sufficient number of general practitioners within the Morbi government health system, but the district has a severe shortage in the available number of specialists, with only one internal medicine physician, one pediatrician and no obstetricians or gynecologists in the district health administration for the entire population. The program was implemented in Maliya and Tankara blocks, which were the hard-to-reach parts of the district, with a patient population with poorer health and socioeconomic indicators than the district median and the median for Gujarat. Frontline health providers would frequently refer patients needing consultations with gynecologists, pediatricians or dermatologists to the nearby district of Rajkot. With this background, considering the shortage of specialist doctors, the use of telemedicine is justifiable in Morbi district.

#### Study participants

The nurses were from the Community Health Officer (CHO) cadre within the District Health public health system. All the CHOs had a Bachelor’s degree in Nursing and had completed the pre-service education program for Community Health Officers from the Ministry of Health’s National Health Systems Resource Center. All CHOs who were participating in the telemedicine program were included in the study.

We trained 22 nurses and 2 supervisors in the use of the telemedicine application and Ayu in a 1.5-day in-person training session in September 2022 in Morbi, Gujarat. The nurses installed the MyTeleDoc application on their personal smart phones by downloading it from the Google Play Store. Each nurse was given 30 standardized patient cases from different clinical specialties to evaluate their proficiency. We asked the nurses to upload at least five cases each and at least one case of each specialty. These standardized patient cases were representative of the morbidity profile of rural primary care clinics in India and included the most seen problems for which the nurses would have to facilitate teleconsultations. The nurses uploaded the evaluation cases using the MyTeleDoc app (white-labeled version of Intelehealth) between September and November 2020.

The user-generated clinical notes were provided to a study team member, who was a trained telemedicine doctor (B.B.), who provided a differential diagnosis and triage decision based on the information provided by the user. Then the doctor was provided the original standardized patient case from which the nurse created the clinical note and asked to compare the nurse’s generated clinical note to the original patient case. We compared the differential diagnosis and triage decision that was arrived at using the information provided by the nurse with the diagnosis and triage decision for the original case. In addition, we also compared how accurately the user-generated clinical note matched the original case. A single physician, who was also the trainer, evaluated the cases. After seeing the case, the evaluating physician did not communicate with the nurse but based their triage decision and diagnosis on the information in the generated clinical note alone.

The following data points were analyzed:

(1) The overall accuracy of the clinical note, i.e. how accurately the generated medical record resembled the patient case study, expressed as a scale from 0 to 10, 0 being completely inaccurate and 10 being completely accurate. The accuracy score is a subjective evaluation metric and a composite of precision and recall.
(2) Percentage of cases with missing information, the importance of the missing information and its impact on the consultation outcome
(3) Correct diagnosis rate
(4) Correct triage rate

We used an accuracy score rather than deductive coding in order to measure both the precision and the recall. This is because we also want to minimize the collection of irrelevant information in a real-world setting by nurses. Unlike in phase 1, the nurses also input information as free text into the qualitative and open-ended questions that were skipped in phase 1. While recall is more important than precision, it is better to have too much information than miss out important information; if too much irrelevant information is gathered by the nurse, it adds to their workload as well as that of the remote doctor.

The data were anonymized to minimize bias. We conducted the data analysis using SPSS. Institutional review board (IRB) approval was obtained from the Johns Hopkins Medicine Institutional Review Board (IRB00144748 and IRB00172154).

#### Acceptability

After the training, as part of the monitoring and evaluation of the program, the nurses were provided a survey developed based on the Technology Acceptance Model (TAM) to assess their attitudes towards this technology. The TAM is a theoretical framework that postulates that the acceptability of a technology solution is dependent on its (1) perceived usefulness, (2) perceived ease of use, (3) the habits of the user, (4) compatibility with their daily workflows, (5) attitude towards technology, (6) facilitating aspects in the organization/environment (facilitators), (7) subjective norms and (8) intention to use the technology. This tool assesses baseline comfort with technology in general and comfort with a specific technology. We developed a questionnaire with 33 questions divided into the eight theoretical dimensions. Questions are answered on a 7-point Likert scale from ‘totally disagree’ (−3) to ‘totally agree’ (+3), and scores are calculated using the mean of the items that constitute each theoretical dimension. We collected demographic data, including age, sex, qualifications and years in clinical practice. This questionnaire was a modified version of an existing validated questionnaire developed by Gagnon *et al*. [23] adapted to our use case. The quantitative survey data were analyzed using Excel.

#### Rationale for the use of standardized patient case studies

The goal of this study was to complete a preliminary ‘bench test’ of Ayu in a non-clinical environment. The use of Standardized Patient Case studies has certain advantages—it is possible to test the system on a wider range of morbidities in the shorter time frame, including health issues with low prevalence. It also provides a reference gold standard against which to benchmark the system performance. Since we used standardized patient case studies and did not conduct the study with actual patients, the risks in the study are minimized and we can evaluate system performance in a low-risk setting prior to deploying with actual patients. The limitations of this methodology are that these cases are well structured and information-rich including the presenting complaint, physicial exams, medical history including allergies, etc., whereas patient-presented histories may not be as structured or information-rich. The study aims to determine if it is an effective tool that can be used in telemedicine settings in developing countries, identify ways to improve it and is a precursor to more rigorous large-sample studies in real-world settings.

## RESULTS

### Evaluation of information retrieval ability

The 190 standardized patient case studies were grouped into the following sub-specialties: infectious diseases (*n* = 54), gastroenterology (*n* = 28), cardiology (*n* = 16), general and family medicine (*n* = 15), dermatology (*n* = 13), pulmonology (*n* = 12), endocrinology (*n* = 10), nephrology (*n* = 8), pediatrics (*n* = 8), neurology (*n* = 7), gynecology (*n* = 6), hematology (*n* = 6), orthopedics (*n* = 5) and ophthalmology (*n* = 2). The cases were for diseases with high (*n* = 101), moderate (*n* = 46) or low (*n* = 43) prevalence in a rural primary care setting. Physicians with experience practicing in primary care settings in rural Gujarat, and specifically in Morbi district, determined the prevalence of the diseases and categorized the cases into High, Moderate and Low prevalence. Prevalence is a measure of how common a disease process is found in a specified at-risk population at a specific time point or during a specified period. For this study convenience, a high prevalence is a disease condition that has very frequent occurrence in a specified time within the study population; low prevalence is a disease condition that has rare occurrence in a specified time within the study population; medium prevalence is a disease condition that is noted sometimes, but neither too common nor rare, in a specified time within the study population. Hence, there is likely some subjectivity in this determination. Table 1 summarizes the results of the study.

**Table 1:** Mean recall for patient history and physical exams

	**Mean recall ± SD (Patient history)**	**Mean recall ± SD (Physical exam)**	**Correct triage rate**	**Correct diagnosis rate**
Overall	0.65 ± 0.19 (*n* = 190)	0.42 ± 0.28 (*n* = 174)	88%	68%
				
High	0.64 ± 0.22 (*n* = 101)	0.44 ± 0.29 (*n* = 92)	92%	92%
Moderate	0.62 ± 0.16 (*n* = 46)	0.41 ± 0.28 (*n* = 44)	89%	48%
Low	0.71 ± 0.15 (*n* = 43)	0.38 ± 0.26 (*n* = 38)	79%	35%
				
Infectious diseases	0.62 ± 0.21 (*n* = 54)	0.46 ± 0.28 (*n* = 50)	87%	78%
Gastroenterology	0.63 ± 0.16 (*n* = 28)	0.40 ± 0.26 (*n* = 27)	89%	43%
Cardiology	0.70 ± 0.18 (*n* = 16)	0.44 ± 0.32 (*n* = 16)	75%	69%
General medicine	0.67 ± 0.18 (*n* = 15)	0.28 ± 0.33 (*n* = 11)	100%	93%
Dermatology	0.64 ± 0.28 (*n* = 13)	0.46 ± 0.36 (*n* = 13)	77%	77%
Pulmonology	0.60 ± 0.07 (*n* = 12)	0.50 ± 0.13 (*n* = 12)	83%	17%
Endocrinology	0.56 ± 0.19 (*n* = 10)	0.29 ± 0.09 (*n* = 10)	90%	100%
Nephrology	0.58 ± 0.13 (*n* = 8)	0.38 ± 0.12 (*n* = 7)	88%	75%
Pediatrics	0.72 ± 0.15 (*n* = 8)	0.53 ± 0.25 (*n* = 6)	100%	50%
Neurology	0.69 ± 0.19 (*n* = 7)	0.47 ± 0.29 (*n* = 6)	100%	71%
Gynecology	0.90 ± 0.13 (*n* = 6)	0.61 ± 0.54 (*n* = 3)	100%	50%
Hematology	0.84 ± 0.08 (*n* = 6)	0.41 ± 0.25 (*n* = 6)	83%	83%
Orthopedics	0.64 ± 0.19 (*n* = 5)	0.19 ± 0.24 (*n* = 5)	100%	100%
Ophthalmology	0.54 ± 0.35 (*n* = 2)	0.36 ± 0.51 (*n* = 2)	100%	50%

The number of presenting complaints in a case ranged from one to seven complaints, with an average of three presenting complaints per case. Overall, the mean and standard deviation of the recall score was 0.65 ± 0.19 (*n* = 190) for the patient history and 0.42 ± 0.28 (*n* = 174) for the physical exam. Sixteen cases were excluded from the calculation of the recall score for the physical exam since those case studies did not contain any physical exam information. Thus, on average, Ayu successfully captured 65% of patient history information and 42% of physical examination information.

The overall recall for the patient history was higher than for physical exams (see Table 1). The higher recall for history is understandable since several physical exams cannot be task-shifted to a nurse or a community health worker. Notable physical exam information missed due to inability to task-shift included exams related to auscultation, percussion and palpation of the chest or abdomen. Neurological examination information also could not be task-shifted.

We observed that the information collected using the system was sufficient to arrive at the correct triage decision in 88% (*n* = 190) of cases and sufficient to arrive at the correct diagnosis in 68% (*n* = 190) of cases.

A noteworthy observation was that the information was sufficient to arrive at the correct diagnosis in 92% (*n* = 101) of cases with high prevalence, 48% (*n* = 46) of cases with moderate prevalence and 35% (*n* = 43) for cases with low prevalence. We observed a statistically significant association between the diagnostic accuracy and prevalence of the case (*P* < 0.05, chi-square test). However, the mean recall score for the patient history did not vary much between high-, moderate- and low-prevalence cases (0.64, 0.62 and 0.71, respectively). Similarly, the mean recall score for physical exams did not vary much between high-, moderate- and low-prevalence cases (0.44, 0.41 and 0.38, respectively), indicating that Ayu still collected the same proportion of information irrespective of the prevalence of the case. A Kruskal–Wallis H test further confirmed that the differences in mean recall scores were not statistically significant with χ^2^(2) = 5.007, *P* = 0.082 for recall of history and χ^2^(2) = 1.008, *P* = 0.604 for recall of physical exam information.

**Figure 2:**
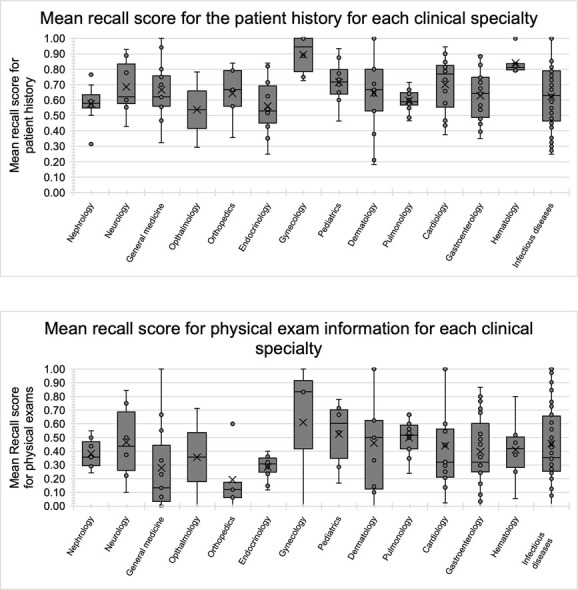
Mean recall score for each clinical specialty

Gynecology cases have the highest mean recall score (0.90) for the patient history, and Ophthalmology has the lowest mean recall (0.54), see Table 1 and Fig. 2. A Kruskal–Wallis H test showed a statistically significant difference in the recall score for the patient history between the 14 different clinical specialties, χ^2^(2) = 24.398, *P* = 0.028. However, a *post hoc* analysis using Dunn’s method revealed no significant differences between the pairwise groups (*P* < 0.05). This difference is likely due to the low sample size in some specialties and the global *P*-value being relatively close to the desired significance level.

Gynecology cases again have the highest mean recall score (0.61), and Orthopedics has the lowest mean recall score (0.19) for physical exams, see Table 1 and Fig. 2. However, a Kruskal–Wallis H test showed no statistically significant difference in the recall score for physical exams between the specialties, χ^2^(2) = 14.669, *P* = 0.328. The information retrieval capability of the system with regards to the patient history may vary for different specialties, but the system’s performance in capturing physical examination information appears to be the same across specialties.

### Fidelity of use by nurses

The average age of CHOs was 27 years, with 16 CHOs in the age group of 20–29 and six CHOs in the 30–39 years age group. All the CHOs were posted at their Health and Wellness Centers (HWCs) for less than a year. The CHOs varied in the total number of years of work experience as a nursing professional before becoming a Community Health Officer—10 CHOs had less than a year of work experience, 9 CHOs had 1–5 years of experience, 1 CHO had 6–10 years of experience and 2 CHOs had >10 years of work experience. All CHOs owned personal smartphones and were very comfortable using mobile devices and apps.

**Figure 3:**
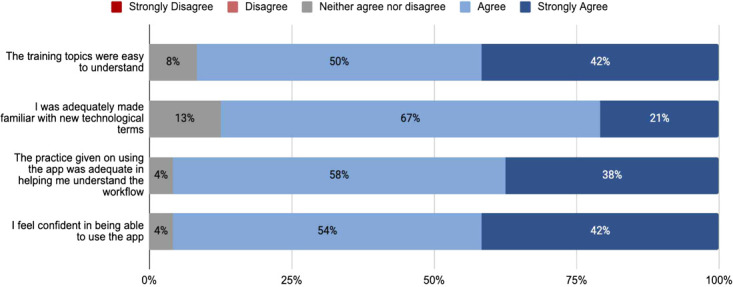
Post-training feedback from users

A structured training curriculum was developed and was delivered over 1.5 days in December 2019. The training included sessions on the use of the app, clinical training in conducting physical exams, telemedicine norms and guidelines and communication skills. Several role plays were conducted during the training to ensure participants were comfortable using the application and the digital assistant. Participants’ post-training feedback showed that they felt confident and proficient in using the application (Fig. 3). Ninety-two percent of training participants rated the training topics as easy to understand. Eighty-eight percent felt they were made adequately familiar with technological terms. Ninety-six percent felt that the training was adequate in understanding the technology and workflows of telemedicine. Ninety-six percent felt confident in being able to use the app. Thus, we may conclude that usability issues in the technology platform may not impact the quality of the teleconsultation.

After the training, three nurses dropped out as they received a transfer to a health facility in another district (13.6% dropout rate). The 19 remaining study participants uploaded 203 evaluation cases.

**Figure 4:**
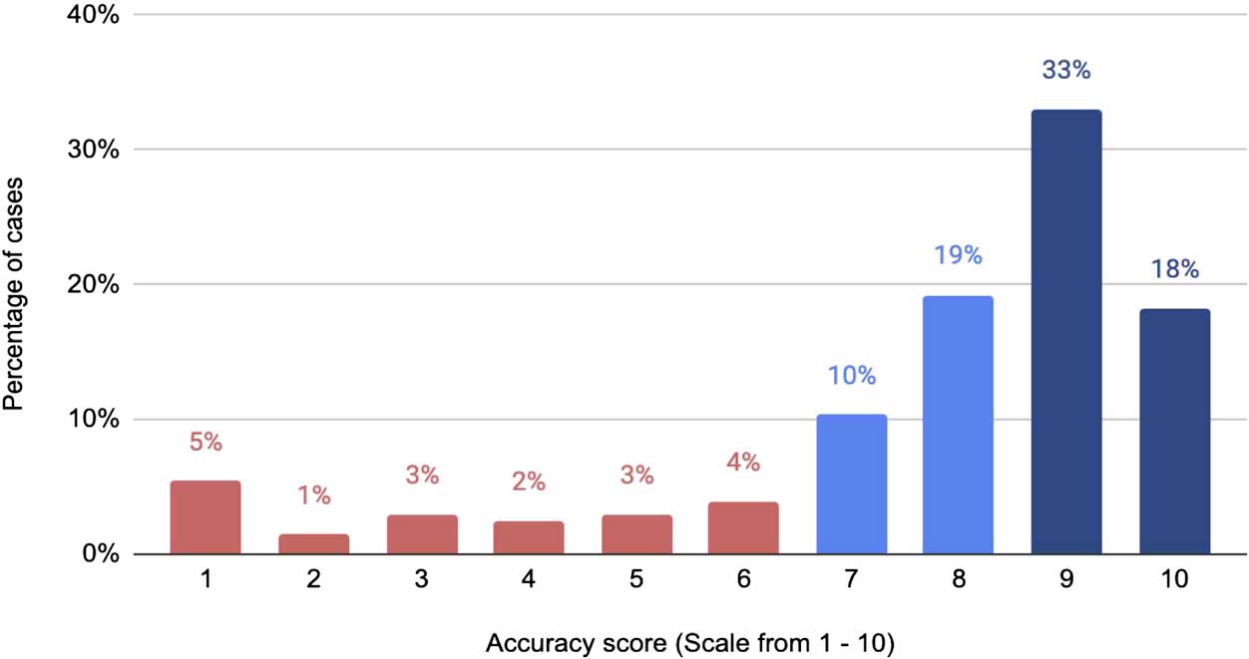
Proportion of cases with high (9 or 10), acceptable (7 or 8) and poor (6 or below) accuracy scores

Out of the 203 cases, we observed that 58% (*n* = 117) cases had no information missing, 17% (*n* = 34) had some information missing that did not impact the diagnosis and 26% (*n* = 52) had important information missing that would have changed the patient’s diagnosis. The mean overall accuracy score of the user-generated clinical note was 7.7, evaluated on a Likert scale from 0 (not at all accurate to the patient case study) to 10 (completely accurate to the patient case study). Nineteen percent (*n* = 39) of cases had a poor score of 6 or lower, 30% (n = 6) had an acceptable score of 7 or 8 and 52% (*n* = 106) had a high score of 9 or 10, see Fig. 4.

**Figure 5:**
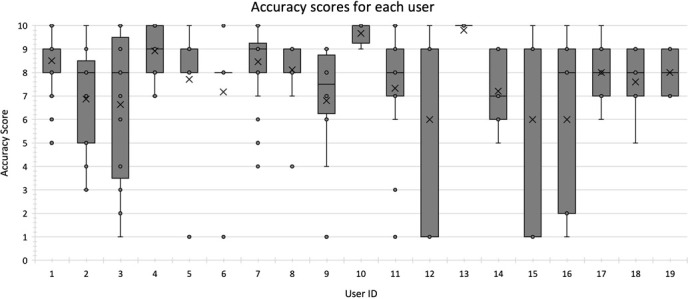
Mean and distribution of accuracy scores across the nurse users

Figure 5 shows the mean and distribution of accuracy scores for each user, and Table 2 gives the mean accuracy score and the number of cases uploaded by each user. A Kruskal–Wallis H test showed a statistically significant relationship between the user and the mean accuracy score, χ^2^(2) = 34.865, *P* = 0.010. However, a *post hoc* test showed no significant differences between the pairwise means.

**Table 2:** Fidelity of use of a digital assistant by nurses

	**No. of clinical notes**	**Mean accuracy ± SD**	**Percent of clinical notes with missing information**	**Correct triage rate**	**Correct diagnosis rate**
Overall	203	7.71 ± 2.42	42%	85%	74%
					
Nurse 1	28	8.46 ± 1.50	21%	93%	89%
Nurse 2	27	7.33 ± 2.50	37%	89%	85%
Nurse 3	24	8.50 ± 1.22	33%	96%	83%
Nurse 4	19	6.63 ± 3.18	63%	74%	63%
Nurse 5	15	6.87 ± 2.29	73%	67%	40%
Nurse 6	13	8.92 ± 1.12	46%	92%	77%
Nurse 7	10	6.80 ± 2.57	80%	70%	50%
Nurse 8	9	8.11 ± 1.69	22%	89%	89%
Nurse 9	7	7.71 ± 3.04	14%	86%	86%
Nurse 10	6	7.17 ± 3.13	50%	83%	83%
Nurse 11	6	9.67 ± 0.52	0%	100%	100%
Nurse 12	5	6.00 ± 4.58	40%	60%	60%
Nurse 13	5	9.80 ± 0.45	0%	100%	100%
Nurse 14	5	7.20 ± 1.79	60%	80%	40%
Nurse 15	5	6.00 ± 4.58	40%	80%	60%
Nurse 16	5	6.00 ± 4.18	40%	60%	60%
Nurse 17	5	8.00 ± 1.58	80%	80%	80%
Nurse 18	5	7.60 ± 1.67	80%	100%	60%
Nurse 19	4	8.00 ± 1.15	50%	75%	50%

The generated clinical notes could be triaged into the following categories: ‘remote management over telemedicine’, ‘urgent referral to a facility’, ‘non-urgent referral to a facility’ or ‘could not triage due to insufficient information’. Of the 203 cases, 91% (*n* = 185) of the cases could be correctly triaged, 9% (*n* = 18) were incorrectly triaged and 6% (*n* = 13) of cases could not be triaged due to insufficient information in the user-generated clinical note. Based on the generated clinical note, the correct diagnosis was provided in 74% (*n* = 153) of cases, a partially correct diagnosis was provided in 10% (*n* = 20) of cases, an incorrect diagnosis was provided in 13% (*n* = 26) of cases and no diagnosis could be provided in 3% of cases (*n* = 6). There was a strong correlation between the accuracy score and correct diagnosis (*P* < 0.00001, unpaired *t*-test assuming equal variances). Table 2 summarizes the results of this section of the study.

### Evaluation of acceptability

**Figure 6:**
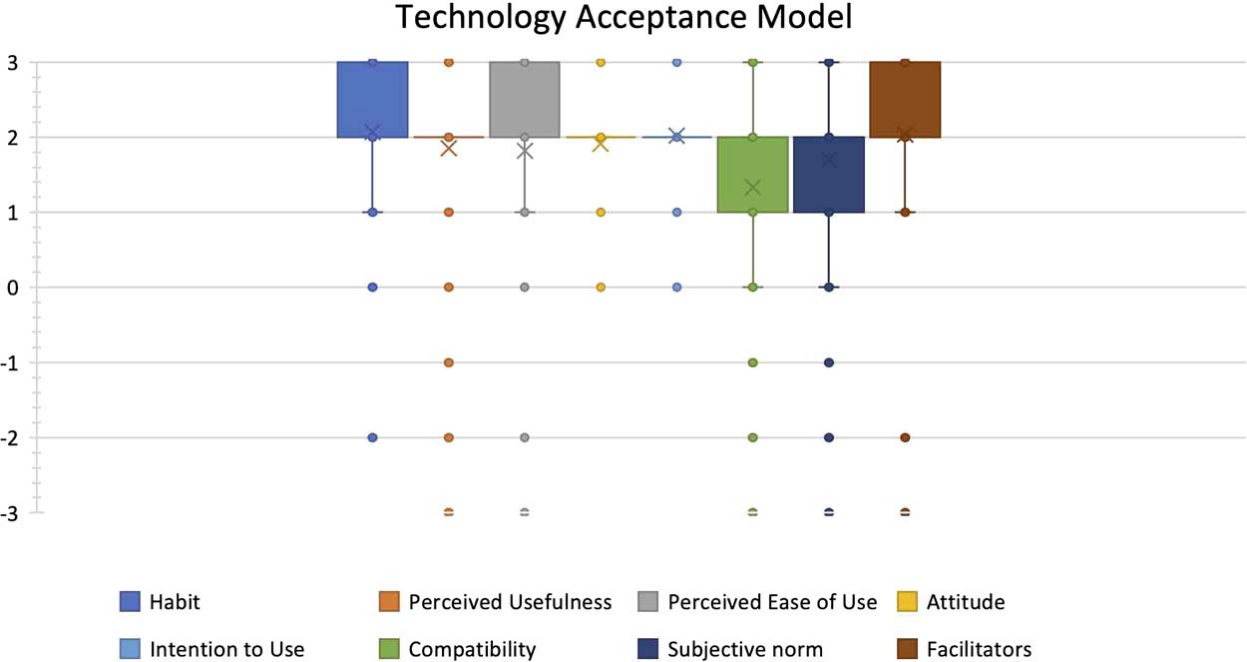
Technology acceptance model scores

Nurses rated the solution on various aspects of the TAM on a 7-point Likert scale from Totally disagree (−3) to Totally agree (+3). The overall Chronbach’s alpha for the survey was 0.94, indicating the high reliability of the survey instrument. Users reported that they perceived the solution to be useful (mean score for perceived usefulness: 1.85), easy to use (mean score for perceived ease of use: 1.82) and compatible with their daily workflows (mean score for compatibility: 1.33). They felt that patients, colleagues and supervisors would positively view them using the system (mean score for subjective norms: 1.71). They reported a high level of availability of facilitating aspects in the organization/environment such as availability of devices, power and internet (mean score for facilitators: 2.04) and that they were already well habituated to using technology (mean score for habits: 2.07). They expressed a high intention to use the system (mean score for intention to use: 2.02) and a positive attitude towards the impact of the system (mean score for attitude: 1.91). Overall, frontline health workers showed high acceptability towards the system (see Fig. 6).

The first component of our evaluation only looked at the information retrieval ability of the digital assistant by an ‘ideal user’ on a wide range of cases, which was found to be modest. The second component of our evaluation explored the information retrieval ability of the digital assistant in the hands of the intended user group within a smaller range of cases, where again the results were acceptable. Finally, we examined the system’s acceptability in the intended user group, which was high.

## DISCUSSION

The value of a digital assistant for history-taking in our context of telemedicine-based primary health care in resource-limited settings can best be understood by examining its ability to improve, or at the bare minimum, comply with the standard of care that patients should receive in such a setting. It is difficult to estimate the current quality of history-taking and physical examination in resource-limited settings, whether in primary care or telemedicine. Very few studies that examine these questions have been conducted. A study shows that the average primary care consultation in India lasts 2.5 minutes [24] and that in primary care clinics, licensed health care providers only completed between 16 and 22% of essential history-taking tasks [25,26]. Our results show that using an assisted history-taking system can reduce the time burden on the physician and improve diagnostic accuracy by providing them the evidence-based history upfront.

This is important in a telemedicine context where the medical history is often the only source of diagnostic information. Schoenfeld *et al.* used 67 standardized patients presenting with common acute illnesses (such as viral pharyngitis, acute rhinosinusitis, low back pain) who underwent 599 virtual visits and found that diagnoses were correctly named in 69.6% of visits across eight websites [2]. The wrong diagnosis was provided in 14.8% of visits, and no diagnosis was provided in 8.7% of visits [2]. The diagnostic accuracy varied by condition and by telemedicine company [2]. The same study found that completeness in history-taking and physical examination in virtual visits varied from 51.7 to 82.4% based on the Direct-to-Consumer (DTC) telemedicine provider [2].

In comparison, our study showed a 74% diagnostic accuracy. On average, Ayu captured 65% of clinical information in the medical history and 42% of clinical information from physical exams. Thus, the overall system performance is modest, and there is more score for future development. Both the proportion of captured information in the medical history and via physical exams can be increased. However, there is a limit to what can be safely and effectively task-shifted to frontline health workers. This would largely depend on the qualifications and clinical skills of the health worker—for example, nurses will be able to perform a wider range of physical exams than a community health worker. In the absence of any kind of standards in the appropriate amount of information that should be gathered during a patient assessment, the most important measure of whether the proportion of history and physical exam information is appropriate is by observing its impact on the diagnostic outcome as a result of this information. We plan to address this in future studies.

Recent studies have shown that assisted history-taking systems can improve the quality of the differential diagnosis provided [27,28]. In 68–74% of cases, the information provided by Ayu was sufficient to arrive at the correct diagnosis, with this proportion being higher in certain specialties. In the most prevalent cases, Ayu’s information was sufficient to arrive at the correct diagnosis in 92% of cases. While the history is certainly important, there will be patients for whom additional diagnostic testing and referrals are indicated. The teledoctor cannot provide a provisional diagnosis for a certain proportion of patients, and referral is essential. We observed that the type of health issue has a significant relationship with the quality of the history note, indicating that certain types of health issues in primary care, especially those that rely more on the verbal history, are better suited to telemedicine. In contrast, issues in orthopedics that rely more on physical examination are not amenable to telemedicine. An essential change in healthcare practice for physicians who are consuming the output note of Ayu is to be careful in their diagnostic reasoning and not rely entirely on the output note. The physician must carefully judge if the information provided is sufficient and ask further probing questions since several aspects of history-taking and physical exams cannot be task-shifted. It falls upon the remote physician to gather this information and complete the clinical note.

We observed a strong relationship between the amount of information captured during history-taking and the diagnostic accuracy, i.e. cases where more information was collected were more likely to be correctly diagnosed. Increasing the recall score of the digital assistant through more in-depth questioning in future versions of Ayu could positively impact the diagnostic accuracy. The recall score, and hence the diagnostic accuracy, was higher for some specialties than others for the medical history. A relatively high or low recall score for a particular clinical specialty may indicate that when Ayu was developed, the specialists that were involved may have been more skilled in certain specialties, which resulted in a better score for these. Specialties with a lower recall score should be prioritized for additional review in future versions of Ayu.

The use of Ayu could enable a more efficient teleconsult by task-shifting some of the essential history-taking steps to a frontline health worker. Given the enormous time constraints and high patient burden placed on primary care physicians, it is safe to assume that this task-shifting could only improve adherence to the standard of care. Several studies have also shown that the outputs of assisted history-taking systems are comparable to or can even be superior to a physician-collected history [17,19, 29]. One of the main drawbacks of the other systems in the literature that we investigated is that they promote perfect information with extensive history-taking assistants with thousands of questions and exhaustive lists. This results in lower acceptability by the system’s users, who might find it too time-consuming to use. It also places a burden on the system developer to maintain such an extensive system. One of the advantages of Ayu is that it allows for the capture of a patient’s history without the expenditure of a physician’s time; however, it does come at the expense of the frontline health workers’ time. In a setting like India, FHWs, while less busy than doctors, are still also very busy health professionals. An important consideration is the acceptability of the frontline health worker to take on this new role, given the already high burden of work that falls onto frontline health professionals. Our study indicates high acceptability to take on this role. The needs of the frontline health professional and minimizing the demands on their time are vital for responsible task-sharing. In the current version of Ayu, an FHW, on average, requires 7–10 minutes to collect the patient history. We felt that increasing the information collection burden would significantly reduce the acceptability and intention to use the system in our interactions with nurses.

Thus, this becomes a value-of-information problem. It is difficult to collect this information from a patient directly in a resource-limited setting due to their challenges in navigating a digital interface. It thus falls upon the frontline health worker to collect this information. We found a clear correlation between user error and the quality of diagnosis. Hence, proper user training for the frontline health professional is important. We observed a mean accuracy score of 7.71 when used by nurses, indicating that the information-gathering ability of the assistant is reasonably good, even in the hands of the intended user group. This already saves a considerable amount of the physician’s time, allowing them to focus their efforts on confirming essential information and collecting additional data to confirm their diagnostic hypothesis. Increasing the accuracy score through better user training should be addressed in future work.

Another factor impacting the diagnostic accuracy was the prevalence of the case. Even though the recall scores for cases with high, moderate and low prevalence were not significantly different (0.64, 0.62 and 0.71, respectively, for the patient history and 0.44, 0.41 and 0.38, respectively, for the physical exams), the diagnostic accuracy was very different for each (92, 48 and 35%). The correct diagnosis rate may not be a function of the information-gathering ability of the assistant but rather the diagnostic reasoning of the physician who is trained in thinking about the most likely diagnosis. This is often referred to by the aphorism ‘When you hear hoof beats, think horses, not zebras’. Thus, the results of this study should be interpreted keeping in mind the impact of prevalence on the correct diagnosis rate. Coupling the output of the history note with a differential diagnosis generator or diagnostic checklists could improve the diagnostic reasoning of the physician [30,31].

### Limitations and future work

An important limitation of this study is that it was not performed with actual patients. Thus, aspects of interpersonal communication and trust related to the quality of history-taking are missed. The nurses who participated in the study are trained in a patient-centered manner. Since they practice in the local communities, they may be more acquainted with local customs and social norms, resulting in a more effective communication experience than if the patient were to speak with a teledoctor on a video call directly. On the other hand, the doctor’s persona, gravitas and experience can generate a greater sense of trust in the patient, and doctors may be able to elicit better the more salient aspects of history such as body language, demeanor and tone to guide their questioning. Overall, the use of the assistant does not replace the role of a doctor in the healthcare consult. Digital tools cannot replace humans, but they can relieve some of their burdens. While efforts were made to minimize bias through data anonymization of evaluators to the nurses being evaluated, it would be impossible to eliminate it.

The use of standardized patient case studies instead of actual patients also limits our ability to draw conclusions about the efficacy of Ayu. We have also conducted additional studies with patients in outpatient primary care settings comparing the history notes generated by experts with those generated by a nurse with Ayu as well as comparing the diagnostic decision of an in-person physicial consult with that of a teledoctor consultation enabled by a nurse using Ayu [32].

Further work is planned to improve the comprehensiveness of data collection of this assisted history-taking system. This study has provided important data on what information was missed from case studies, and these findings are being used to improve the questions in Ayu. We also plan to conduct future studies to identify and improve the precision of Ayu. We will use feedback from the nurses as well as from users at other implementation sites that use the Intelehealth software and Ayu to improve its usability and reduce the time taken to complete a guided history-taking encounter.

## CONCLUSION

Ayu, an assisted history-taking system, can enable the collection of a preliminary history of presenting illness, medical history, family history and physical examination through a community health professional at the bedside of the patient and collect at least 65% of essential history information and 42% of essential physical exam information with an overall quality score of 7.71. Ayu’s information was sufficient to arrive at a diagnosis in 92% of cases in the most prevalent cases. The system was acceptable to nurses, who also showed a high intention to use the system. Further work is required to evaluate the system using actual patients. Collection of a proper medical history by a frontline health worker through task-shifting aided by a digital assistant, followed by a remote physician asking appropriate follow-up questions, may improve the quality of history-taking and thus improve diagnostic accuracy in teleconsultations.

## ACKNOWLEDGEMENTS

We thank Dr. Deepak Langade (DY Patil University, Navi Mumbai) for his contributions to the design of the study and compliance with ethical norms in the conduct of human subjects research. We thank Gaurav Srivastava for contributing to the statistical analysis of the data. We would like to thank Dr. Dipannita Kaushik for participating in the data collection and analysis.

All persons who have made substantial contributions to the work reported in the manuscript (e.g. technical help, writing and editing assistance, general support), but who do not meet the criteria for authorship, are named in the Acknowledgements section and have given us their written permission to be named. If we have not included an acknowledgement, then that indicates that we have not received substantial contributions from non-authors.

## CONFLICT OF INTEREST STATEMENT

N.V. is a founder of and serves as the CEO of Intelehealth, a 501(c)(3) non-profit supporting the development and implementation of the telemedicine software. S.A. is a founder of and serves as the Board President of Intelehealth. N.V. and S.A. are also the inventors of the technology involved in the Intelehealth app, which was used in the study described in this publication. This arrangement has been reviewed and approved by the Johns Hopkins University in accordance with its conflict of interest policies.

## AUTHORS' CONTRIBUTIONS

All persons who meet authorship criteria are listed as authors, and all authors certify that they have participated sufficiently in the work to take public responsibility for the content, including participation in the concept, design, analysis, writing or revision of the manuscript. Furthermore, each author certifies that this material or similar material has not been and will not be submitted to or published in any other publication before its appearance in Telemedicine & eHealth.

##  

*Category 1.*

Conception and design of study: N.V., B.B., R.S.P., R.T., I.M., H.R., J.M.K., S.A.

Acquisition of data: N.V., B.B., R.S.P., R.T., I.M., H.R.

Analysis and/or interpretation of data: N.V., B.B., R.S.P., R.T.

*Category 2.*

Drafting the manuscript: N.V., B.B., R.T., S.A.

Revising the manuscript critically for important intellectual content: N.V., B.B., R.S.P., R.T., I.M., H.R., J.M.K., S.A.

*Category 3.*

Approval of the version of the manuscript to be published: N.V., B.B., R.S.P., R.T., I.M., H.R., J.M.K., S.A.

## FUNDING STATEMENT

Not applicable.

## DATA AVAILABILITY STATEMENT

The data underlying this article cannot be shared publicly for the privacy of individuals that participated in the study.
